# HIV Point-of-Care Testing in Canadian Settings: A Scoping Review

**DOI:** 10.3389/fpubh.2017.00076

**Published:** 2017-04-18

**Authors:** Alexa Minichiello, Michelle Swab, Meck Chongo, Zack Marshall, Jacqueline Gahagan, Allison Maybank, Aurélie Hot, Michael Schwandt, Sonia Gaudry, Oliver Hurley, Shabnam Asghari

**Affiliations:** ^1^Centre for REACH in HIV/AIDS, Toronto, ON, Canada; ^2^Health Sciences Library, Memorial University of Newfoundland, St. John’s, NL, Canada; ^3^School of Health Sciences, University of Northern British Columbia, Prince George, BC, Canada; ^4^Division of Community Health and Humanities, Faculty of Medicine, Memorial University of Newfoundland, St. John’s, NL, Canada; ^5^Department of Social Development Studies and School of Social Work, Renison University College, University of Waterloo, Waterloo, ON, Canada; ^6^Health Promotion Division, Faculty of Health Professions, School of Health and Human Performance, Dalhousie University, Halifax, NS, Canada; ^7^Center for Rural Health Studies, Primary Health Care Research Unit, Department of Family Medicine, Memorial University of Newfoundland, St. John’s, NL, Canada; ^8^Coalition des organismes communautaires québécois de lutte contre le sida, Montreal, QC, Canada; ^9^Faculty of Community Health and Epidemiology, College of Medicine, University of Saskatchewan, Saskatoon, SK, Canada; ^10^CIHR Centre for REACH in HIV/AIDS and CIHR CBR Collaborative: A Program of REACH, Toronto, ON, Canada

**Keywords:** HIV, point-of-care testing, utilization, Canada, scoping review

## Abstract

**Background:**

HIV point-of-care testing (POCT) was approved for use in Canada in 2005 and provides important public health benefits by providing rapid screening results rather than sending a blood sample to a laboratory and waiting on test results. Access to test results soon after testing (or during the same visit) is believed to increase the likelihood that individuals will receive their results and improve access to confirmatory testing and linkages to care. This paper reviews the literature on the utilization of HIV POCT across Canadian provinces.

**Methods:**

We searched OVID Medline, Embase, EBM Reviews, PsycINFO, CINAHL, and 20 electronic grey literature databases. All empirical studies investigating HIV POCT programs in Canada published in French or English were included.

**Results:**

Searches of academic databases identified a total of 6,091 records. After removing duplicates and screening for eligibility, 27 records were included. Ten studies are peer-reviewed articles, and 17 are grey literature reports. HIV POCT in Canada is both feasible and accepted by Canadians. It is preferred to conventional HIV testing (ranging from 81.1 to 97%), and users are highly satisfied with the testing process (ranging between 96 and 100%).

**Conclusion:**

The majority of studies demonstrate that HIV POCT is feasible, preferred, and accepted by diverse populations in Canada. Losses to follow-up and linkage rates are also good. However, more research is needed to understand how best to scale up HIV POCT in contexts that currently have very limited or no access to testing.

## Introduction

HIV testing and diagnosis is the first stage in the HIV continuum of care. Previous studies on HIV-infected individuals suggest that people who are aware of their HIV status are more likely to practice behaviors that lower the risk of HIV transmission, compared to people who are unaware of their HIV status (1). Public health practitioners recommend widespread availability and accessibility of HIV point-of-care testing (POCT) tests, particularly for priority populations (2).

Globally, HIV POCT has been available for use in recent years. Although a low-cost and easy-to-use test such as HIV POCT has great potential for advancing the UNAIDS 90–90–90 targets; the adoption, implementation, and performance of HIV POCT in practice has proved challenging (3). A systematic approach at the national level including the development of proper policies, regulations, and guidelines related to HIV POCT and a stepwise approach including attention to implementation have been recognized as key factors in improving HIV testing and diagnosis rates (4). However, to ensure sustainable quality testing, it is important to recognize the challenges in different settings particularly in relation to regulatory control and quality monitoring (3, 5). The Global Health Strategy on HIV/AIDS has put a strong emphasis on monitoring interventions across the entire continuum of care.

HIV POCT has been approved in Canada since 2005. To date, POCT programs have been implemented primarily in large Canadian cities such as Vancouver, Montreal, Toronto, Edmonton, Winnipeg, and Saskatoon. POCT refers to the practice of providing a rapid preliminary test result within one clinical encounter, rather than sending a blood sample to a laboratory and waiting on test results. HIV POCT provides an important public health benefit for the estimated one quarter of Canadians living with HIV who are unaware of their HIV status (6). This benefit is twofold. First, HIV testing significantly improves the likelihood that clients will receive a preliminary HIV diagnosis as results are conveniently available within minutes of testing (7, 8) Second, it can help to facilitate timely linkages to treatment and care as clients receiving a reactive result are provided with posttest counseling and referrals to care (9, 10). Unlike standard HIV tests, HIV POCT can be performed in any place and has the potential to be more patient-centered and support person-first care (4).

In Canada the only POCT test available: the INSTI HIV-1/HIV-2 Antibody Test has high sensitivity and specificity (>99%). Moreover, current HIV testing guidelines in Canada promote the use of HIV POCT, but these guidelines adhere to strict informed consent and pretest counseling requirements. For example, pretest counseling procedures must clearly convey that test results will be made available within minutes. Moreover, individuals tested must know that results are preliminary, and that confirmatory testing is required for a reactive or indeterminate result (11, 12). HIV POCT programs in Canada will be aware of these guidelines and be required to adapt testing policies accordingly.

The focus of this scoping review was to investigate the utilization of HIV point-of-care-testing in Canadian settings. We sought to understand what is known about the use and implementation of HIV POCT in Canadian settings and to identify gaps in the current knowledge base. The review describes general characteristics of existing POCT programs in Canada and then synthesizes the relationships between HIV POCT programs and acceptability, satisfaction, preference, feasibility, returned results, losses to follow-up, and linkage to care rates. For the purpose of this scoping review, the following terms were operationalized as:

- Acceptability: the proportion of testers willing to receive or who received a HIV POCT and/or reasons for acceptance.
- Feasibility: a determination that HIV POCT is both easily done and convenient.
- Linkage to care: the proportion or number of people who receive confirmatory positive HIV results and are linked to care.
- Loss to follow-up: the proportion or number of people tested who receive a reactive POCT but do not receive western blot confirmatory testing results.
- Preference: the proportion of testers who favored POCT when compared with conventional testing and/or reasons influencing one’s preference.
- Reach: the proportion of individuals who were tested using HIV POCT technology who were previously never or recently tested.
- Returned results: the proportion or number of people who receive their POCT result as compared to the number of people who are tested.
- Satisfaction: the proportion of testers who were pleased with their POCT experience and/or reasons for their satisfaction.
- Sensitivity, specificity and predictive value of HIV POCT.

Previous systematic reviews have investigated the utilization of conventional HIV testing in Canadian settings (13), the barriers associated with HIV POCT in an international context (14), as well as the acceptability of HIV self-testing including participants’ attitudes and testing uptake (15). This scoping review adds to the literature by focusing on the utilization of HIV POCT in Canada.

## Methods

### Search Strategy

The search strategy using a combination of controlled vocabulary and keyword searching was developed to capture literature relating to HIV POCT. See Table 1 for a sample search strategy. As is recommended by Arksey and O’Malley (16), a wide study selection and database search was conducted to generate breadth of coverage on the research topic.

**Table 1:** **Summary of systematic search strategy**.

Search strategy
1. HIV Infections/di [Diagnosis] (12,845)
2. HIV Seropositivity/di [Diagnosis] (2,397)
3. AIDS Serodiagnosis/ (6,158)
4. HIV.ti. (148,826)
5. human immunodeficiency virus.ti. (30,058)
6. or/1–5 (179,146)
7. Point-of-Care Systems/ (7,372)
8. POCT.ti,ab. (588)
9. point of care.ti,ab. (7,517)
10. point of service.ti,ab. (345)
11. ((rapid or instant or home or self) adj3 (test$ or screen$ or kit$)).ti,ab. (28,828)
12. oraquick.ti,ab. (110)
13. clearview.ti,ab. (97)
14. (reveal adj2 rapid).ti,ab. (146)
15. insti.ti,ab. (69)
16. uni-gold recombigen.ti,ab. (7)
17. multispot.ti,ab. (84)
18. (sure adj check).ti,ab. (1)
19. stat-pak.ti,ab. (62)
20. chembio.ti,ab. (29)
21. or/7-20 (40,720)
22. 6 and 21 (2,185)
23. remove duplicates from 22 (2,045)

*Database: Ovid MEDLINE(R) in-process and other non-indexed citations and Ovid MEDLINE(R) <1946 to August 2014>*.

The following electronic databases were searched:

- Ovid MEDLINE, including in-process and other non-indexed citations (1946–third week, August 2014)
- EMBASE (1974–25, August 2014)
- EBM reviews (1991–third quarter 2014)
- PsycINFO (1806–25, August 2014)
- CINAHL (1980–25, August 2014)

No language or date limiters were applied.

To supplement the database search, the review team conducted a search of 19 electronic databases for grey literature. The following sites were searched:

- AIDS Committee of Toronto
- ASO411
- BC Centre for Excellence in HIV/AIDS
- Bibliothèque et Archives nationales du Québec
- Canadian Agency for Drugs and Technologies in Health
- Canadian HIV/AIDS Legal Network
- Canadian Nurses Association
- CATIE and http://sagecollection.ca
- Canadian Health Research Collection
- Canadian Institute for Health Information
- CIHR Social Research Centre in HIV Prevention
- Gay Men’s Sexual Health Alliance
- Google custom search: government documents
- Google Scholar
- Health Nexus
- Health Quality Ontario
- Institut national d’excellence en santé et en services sociaux
- Ontario HIV Treatment Network
- Open Grey
- Public Health Agency of Canada.

In addition to our online search strategy, we hand-searched the reference lists of included articles for additional items of relevance. We also contacted 65 Canadian researchers who are members of the national CIHR Centre for REACH (Research Evidence into Action for Community Health) in HIV/AIDS POCT Working Group. These contacts provided additional grey literature materials as well as further knowledge of ongoing POCT testing programs in Canada.

### Inclusion Criteria and Study Selection

Two members of the scoping review team assessed studies based on information in the title and abstract. As is further recommended by Arksey and O’Malley (16), the inclusion criteria was developed *post hoc* based on increasing familiarity with the literature and applied to each article to determine their relevance in this scoping review. Studies were included if they met the following criteria:

- Empirical study investigating HIV POCT programs, including articles investigating access and uptake to HIV POCT.
- Study published in English or French.

Studies that evaluated HIV POCT performance without providing further information about access to testing or testing uptake were excluded. “Access” refers to information about the point of access including structural factors, setting, location, hours, service provider who is offering testing, funding, cost, and time to test. “Uptake” refers to what happens when people are offered a test and whether or not they accept. This concept includes testing rates but is also about acceptability.

A second eligibility stage was completed whereby two members of the scoping review team assessed articles for inclusion in this Canadian-focused scoping review. Literature from both peer-reviewed journals and grey literature sources were included in this review. Items were included that took place in Canadian locations.

**Figure 1:**
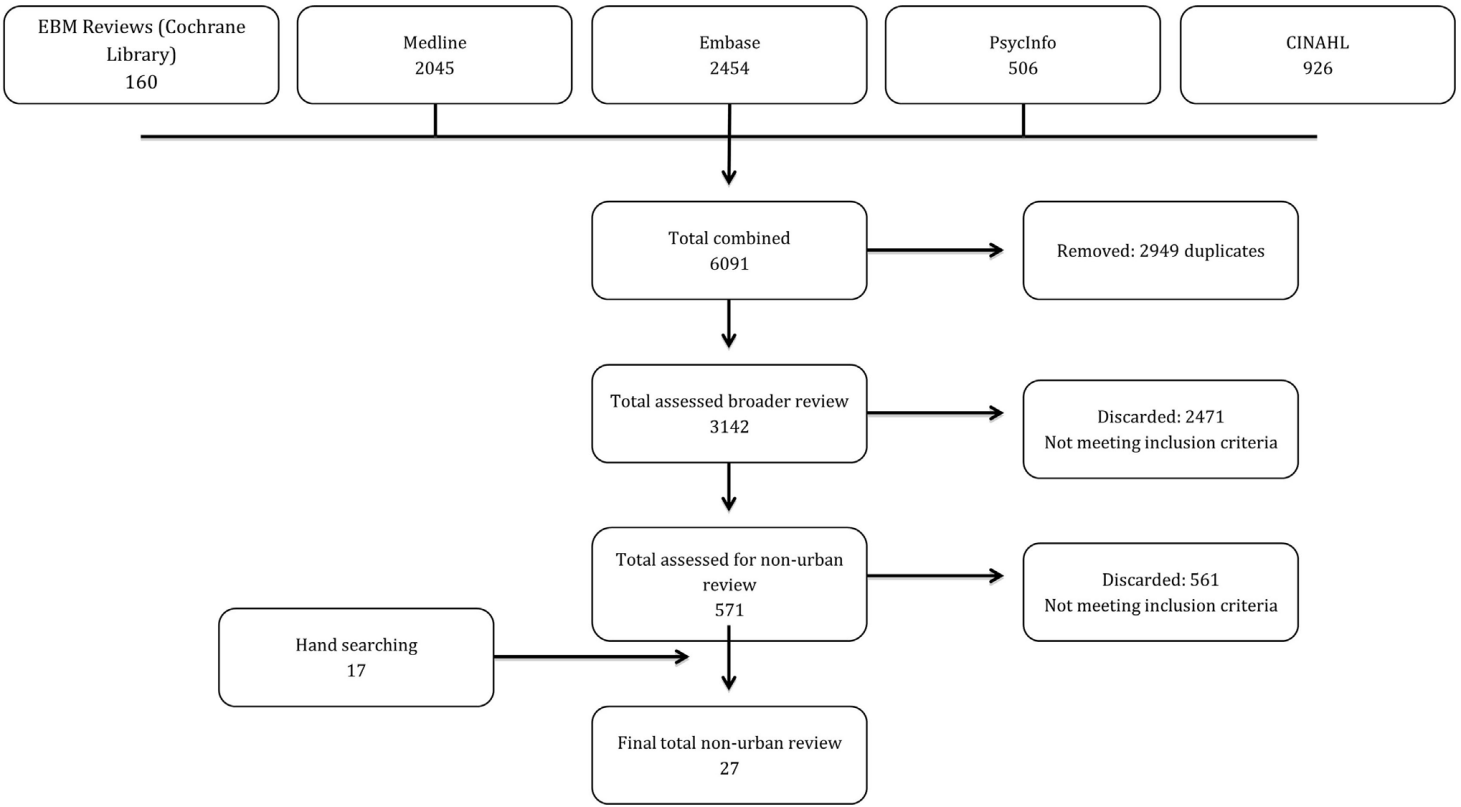
**Flow diagram of studies (PRISMA)**.

The peer-reviewed database search yielded 6,091 records. After duplicates were removed, 3,142 were screened for eligibility resulting in the identification of 571 items of potential relevance to this review. The grey literature search produced an additional 17 articles. Items were typically excluded because they did not focus on HIV POCT, and/or the HIV POCT program described was not located in a Canadian setting. See Figure 1 for the search strategy decision tree.

### Data Extraction and Quality Appraisal

An Excel data extraction sheet, including data extraction guidelines, was prepared to guide the quality appraisal process. Specifically, the tool was designed to organize extracted information relating to citation type, study design and methodology, program participants, and program characteristics such as the test provider, testing combinations, and the site of program delivery. Further information was extracted related to the following outcomes: feasibility, acceptability, preference, satisfaction, and impact including loss to follow-up and linkage to care rates. The data extraction tool was piloted with three articles, revised iteratively, and finalized before the remaining articles were accessed.

Two trained research assistants independently reviewed and extracted the information for each article included in the review. A calibration exercise was undertaken, and eligibility criteria were modified where the agreement between the two reviewers was low (kappa <0.5). The reviewers met biweekly to discuss the extracted information and reach consensus. Discrepancies were adjudicated by a third reviewer.

The quality of each study (both quantitative and qualitative) was also assessed using a scoring system based on the criteria found in “A scoring system for appraising mixed method research, and concomitantly appraising qualitative, quantitative and mixed-methods primary studies in Mixed Studies Reviews” (17). The quality score was not used to exclude studies but rather to identify the overall quality of the evidence base.

## Results and Discussion

### Description of Included Studies

A total of 27 studies met the inclusion criteria. Three articles were written in French, and the remaining documents were written in English. Of the 27 items, 10 were from peer-reviewed journals, and 17 were from grey literature sources. Fourteen studies were quantitative including a mix of cross-sectional (*n* = 7), cohort (*n* = 5), and quasi-experimental designs (*n* = 2). Three studies were qualitative of which two used a narrative design and one used grounded theory. Ten studies used mixed-methods approaches; three were cross-sectional, four were cohort studies, two used participatory action approaches, and one was quasi-experimental (Tables 2 and 3). The review team referenced Creswell’s text (18) on research designs to appropriately categorize the articles by study design.

**Table 2:** **Research methods of included articles**.

Research methods	Total	
	*n* **=** 27	
	*n*	%
Mixed methods	10	37
Qualitative	3	11
Quantitative	14	52

**Table 3:** **Study design of included articles**.

Study design	Total	
	*n* **=** 27	
	*n*	%
Cohort	9	33
Cross-sectional	10	37
Grounded theory	1	4
Narrative	2	7.5
Participatory action	2	7.5
Quasi-experimental	3	11

The 27 articles included in this literature set represent 20 studies that evaluated or described existing HIV POCT programs in Canada, while 7 articles elicited opinions including preferences for HIV POCT in Canadian settings. Sixteen studies surveyed the recipients of HIV POCT, four studies surveyed health-care providers, and seven studies surveyed both recipients and providers of HIV POCT. The majority of studies (50%) were assessed to be of low quality, 25% were of moderate quality, and 25% were assessed as strong. Table 4 provides an overview of all included studies.

**Table 4:** **Overview of included studies**.

Reference	Publication type	Language	Type of test offered	Location	Research focus	Study goals
Becker et al. (27)	Peer-reviewed	English	INSTI HIV-1/HIV-2 antibody test	Winnipeg, Manitoba	HIV point-of-care testing (POCT) program	Evaluate success of program

Bergman et al. (19, 25)	Peer-reviewed	English	INSTI HIV-1/HIV-2 antibody test	Edmonton, Alberta	HIV POCT and syphilis testing program	Evaluate feasibility

Bergman et al. (19, 25)	Grey literature	English	INSTITM HIV-1/HIV-2 rapid antibody test	Edmonton, Alberta	HIV POCT and syphilis testing program	Identify challenges to program implementation

Brondani and Chang (23)	Grey literature	English	HIV POCT—not specified	Vancouver, British Colombia	HIV POCT program	Evaluate acceptability

Bungay et al. (36)	Peer-reviewed	English	HIV POCT—not specified	Western Canada	HIV POCT program	Evaluate preferences and satisfaction

Fielden et al. (31)	Grey literature	English	HIV POCT—not specified	Vancouver and Northern Interior, British Colombia	HIV POCT program	Evaluate preferences

Gahagan et al. (32)	Grey literature	English	No test offered	Halifax, Nova Scotia	Research	Evaluate preferences

Guenter et al. (30)	Grey literature	English	Fast-check HIV-1/2 whole blood (Fast Check, Biochem Immunosystems Inc., Montreal, QC, Canada)	Toronto, Ontario	HIV POCT program	Evaluate satisfaction

Guenter et al. (37)	Peer-reviewed	English	Fast-check HIV-1/2 whole blood	Toronto, Ontario	HIV POCT program	Evaluate satisfaction and predictors of HIV POCT use

Halton Region Health Department (20)	Grey literature	English	HIV POCT—not specified	Milton, Ontario	HIV POCT and STI testing program	Evaluate acceptability and satisfaction

HIV Counselling and Testing Community Advisory Committee, Nova Scotia Advisory Commission on AIDS (21)	Grey literature	English	No test offered	Nova Scotia	Research	Evaluate acceptability

Iqbal et al. (38)	Peer-reviewed	English	No test offered	Toronto, Ontario	Research	Evaluate acceptability

Lambert et al. (35)	Grey literature	French	INSTI HIV-1/HIV-2 antibody test	Montréal, Québec	HIV POCT and hepatitis C (HCV) testing program	Evaluate feasibility, acceptability, satisfaction, preference, reach, and impact

Lewis et al. (33)	Peer-reviewed	English	No test offered	Halifax, Nova Scotia	Research	Evaluate acceptability

Lee et al. (41)	Peer-reviewed	English	INSTITM HIV-1/HIV-2 antibody test	Province-wide, Alberta	HIV POCT program	Performance characteristics of test kits

Miller and Martindale (40)	Grey literature	English	HIV POCT—not specified	Canada-wide	HIV POCT program	Evaluate acceptability, satisfaction, and preferences

Nine Circles Community Health Centre (29)	Grey literature	English	INSTI HIV-1/HIV-2 antibody test	Winnipeg, Manitoba	HIV POCT and STI testing program	Evaluate satisfaction and preferences

Options Clinic (47)	Grey literature	English	INSTI HIV-1/HIV-2 antibody test	London, Ontario	HIV POCT and STI testing program	Determine population served by outreach program

Pai et al. (24)	Peer-reviewed	English	Miriad rapid TP/HBV/HIV/HCV antibody test	Montreal, Quebec	HIV POCT program	Evaluate feasibility and preference

PHS Community Services Society (28)	Grey literature	English	INSTI HIV-1/HIV-2 antibody test	Vancouver, British Colombia	HIV POCT program	Evaluate impact (returned results and linkage to care rates)

Pyra Management Consulting Services Inc. (44)	Grey literature	English	No test offered	Nova Scotia	Research	Understand stakeholder perceptions of POCT

Schwandt et al. (34)	Peer-reviewed	English	No test offered	Not reported	Research	Evaluate preferences

Thériault et al. (22)	Grey literature	French	HIV POCT—not specified	Québec City, Québec	HIV POCT and STI testing program	Evaluate uptake, feasibility, acceptability, and satisfaction

Vancouver STOP Project (46) (dent)	Grey literature	English	HIV POCT—not specified	Vancouver, British Colombia	HIV POCT program	Evaluate acceptability

Vancouver STOP Project (26) (out)	Grey literature	English	HIV POCT—not specified	Vancouver, British Colombia	HIV POCT program	Evaluate impact (returned results and linkage to care rates)

Veillette-Bourbeau (45)	Grey literature	French	INSTI HIV-1/HIV-2 antibody test	Montreal, Québec	HIV POCT program	Describe implementation process

Wertheimer (43)	Grey literature	English	No test offered	Canada-wide	Research	Identify barriers to testing

### Characteristics of POCT Programs in Canada

Of the 20 studies that describe HIV POCT programs in Canada, 12 of the 20 (60%) studies demonstrated the use of rapid finger prick technology, while 8 studies did not specify the exact blood or saliva sampling technology used. Thirteen studies (65%) used HIV POCT technologies alone, while 6 used HIV POCT technologies in combination with STI testing, and 1 study described a multiplex testing strategy whereby POCT technologies were used for HIV, hepatitis C (HCV), and STI testing. In these 20 studies, the majority of tests performed were conducted by nurses (*n* = 11); followed by HIV testing counselors (*n* = 3), outreach workers (*n* = 3), dental professionals (*n* = 2), and community-based researchers (*n* = 1).

In the entire article set (*n* = 27), a number of priority populations were reached. Men who have sex with men (MSM) and lesbian, gay, bisexual, transgender, and queer (LGBTQ) populations were the focus in six and four studies, respectively. People with a history of substance use were a priority population in seven studies, while Aboriginal people were the focus of four, commercial sex workers of five, and incarcerated men and women of two.

### Utilization of HIV POCT in Canada

The included studies described HIV POCT programs currently operating or piloted in the following provinces:

- Alberta (*n* = 3)
- British Colombia (*n* = 7)
- Manitoba (*n* = 2)
- Ontario (*n* = 4)
- Québec (*n* = 4).

For the studies in our review, HIV POCT programs were offered in the following Canadian settings:

- Aboriginal health/friendship centers (*n* = 2)
- Addictions facilities (*n* = 2)
- Community-based organizations (*n* = 5)
- Community health centers (*n* = 4)
- Dental offices (*n* = 3)
- Hospital (*n* = 4)
- Indoor commercial sex markets (*n* = 1)
- Primary care centers (*n* = 1)
- Prisons or correctional facilities (*n* = 5)
- Sexual health/HIV clinics (*n* = 4)
- Street outreach (*n* = 4).

### The Impact of HIV POCT Programs in Canada

The following section focuses on the relationships between HIV POCT and acceptability, feasibility, satisfaction, preference, returned results, losses to follow-up, and linkage to care rates. Table 5 summarizes the findings described below.

**Table 5:** **Summary of findings relevant to utilization of point-of-care testing (POCT) in Canada**.

Reference	Study design	Study setting	Study population	Sample size	Data collection instrument	Feasibility	Acceptability	Satisfaction	Preference
Becker et al. (27)	Cross-sectional	Emergency department at hospital	Emergency department patients	501	Posttest questionnaire and INSTI HIV-1/HIV-2 antibody test	–	–	96% satisfaction	–

Bergman et al. (19, 25)	Cohort	Community health center, bathhouses, gay bars, drop-in center prisons, addictions facilities	Men and women	1,031	INSTI HIV-1/HIV-2 antibody test	81.5%	Highest acceptance among testing sites for MSM and the lowest acceptance at community-based organizations	–	–
			Men who have sex with men (MSM)						
			People who use or have history of injection drug use						
			Commercial sex workers						

Bergman et al. (19, 25)	Narrative	Community health centers, community centers, prisons, drop-in centers	Not reported	Not reported	INSTITM HIV-1/HIV-2 rapid antibody test	–	–	–	–

Brondani and Chang (23)	Cross-sectional	Community dental clinics	Men and women	32	Self-administered questionnaire and HIV test	–	92%	–	–

Bungay et al. (36)	Participatory action research design	Indoor commercial sex markets	Women	113	Survey	–	–	Satisfaction was high for women tested due to flexibility of POCT	POCT preferred as it is less invasive, more comfortable, and less painful than standard test
			Commercial sex workers		Focus group				

Fielden et al. (31)	Cross-sectional	Primary care clinic, sexual health clinic, community health center, hospital, street outreach, aboriginal friendship centers, prisons, dental office, addiction facilities	Men and women, aboriginal peoples	243	Survey	–	–	–	40% preferred POCT to standard
					Interviews				
					HIV test results				

Gahagan et al. (32)	Cross-sectional	Sexual health clinic	Not reported	258	Survey	–	–	–	90% prefer rapid to standard test

Guenter et al. (30)	Cohort	Sexual health clinics	Men and women	1,257	Posttest questionnaire or interview and fast-check HIV-1/2 whole blood test	–	–	98.9% satisfaction (non-reactive testers)	–
								100% satisfaction with reactive testers	

Guenter et al. (37)	Cohort	Sexual health clinic	Men and women	1,257	Posttest questionnaire or interview and fast-check HIV-1/2 whole blood test	–	–	99% satisfaction	–

Halton Region Health Department (20)	Observational	Correctional facilities	Incarcerated men and women	156	Survey		HIV POCT was accepted because results were available immediately	98% satisfaction	
					HIV testing data				

HIV Counselling and Testing Community Advisory Committee, Nova Scotia Advisory Commission on AIDS (21)	Cohort study	Not reported	Men, women, transgender people, aboriginal peoples	50	Interview	–	Acceptability was related to lessening the waiting period, and that rapid testing might be an effective way to reach communities that do not know or do not want to know their HIV status	–	–
			African, Nova Scotians		HIV incidence data				
			PWAs		Policy scan				
			People living with hepatitis C (HCV)						

Iqbal et al. (38)	Cross-sectional	Hospital	Pregnant women	92	Survey	–	59% of women were willing to be tested. Willingness was significantly associated with an interest in learning about HIV treatment options, access to health-care services, and the partner notification process	–	–

Lambert et al. (35)	Before and after	Correctional facilities	Men and women, MSM, people who use injection drugs, commercial sex workers, incarcerated men and women, people from endemic countries	478	Survey	–	72.4%	97.1% satisfaction	93% prefer rapid to standard testing
					Interview				
					HIV testing data				

Lewis et al. (33)	Cross-sectional	Sexual health clinic	Men and women, lesbian, gay, bisexual, transgender, and queer (LGBTQ) individuals	258	Survey	–	–	–	90.3% prefer rapid to standard

Lee et al. (41)	Observational	Hospital	Pregnant women, health-care workers with occupational exposures, acutely ill patients	1,737	INSTITM HIV-1/HIV-2 antibody test	–	–	–	–

Miller and Martindale (40)	Before and after	Not reported	Young gay and bisexual men	300	Survey	–	90%	66% satisfied with testing experience	97% preferred rapid to standard test
					HIV test				

Nine Circles Community Health Centre (29)	Cross-sectional	Community health center	Men and women	54	Survey	–	–	96.6% of clients satisfied with testing experience	Preference for POCT related to benefits of an immediate result
			LGBTQ		Focus group				
			MSM		Document review				
			People who use injection drugs, aboriginal peoples, Asian and African Canadian people, commercial sex workers						

Options clinic (47)	Cohort	Sexual health clinic, youth drop-in center, bathhouses, London Pride, Aboriginal friendship centers, needle exchange programs, university health clinics	MSM	945	Document review	–	–	–	–
			LGBTQ						
			People who use injection drugs, aboriginal peoples, students						

Pai et al. (24)	Cross-sectional	Hospital	Men and women, people who use injection drugs	109	Semi-structured questionnaire and Miriad Rapid TP/HBV/HIV/HCV antibody test	92.4% completion rate	–	–	97.2% preferred multiplex to conventional testing

PHS Community Services Society (28)	Cohort	Community centers, street fairs, single-room occupancy hotels	People who use injection drugs	4,773	Survey	–	–	–	–
					HIV testing data				

Pyra Management Consulting Services Inc. (44)	Narrative research	Not reported	Not reported	22	Interview	–	–	–	–

Schwandt et al. (34)	Cross-sectional	Primary care clinics	Women	100	Self-administered questionnaire	–	–	–	81% prefer rapid to standard

Thériault et al. (22)	Cross-sectional	Sexual health clinics	MSM, people who use injection drugs, commercial sex workers, people who inhale drugs	249	Interviews	Nurses had skills to adopt rapid testing easily into clinical practice	95.4% chose rapid test	All people were either satisfied or very satisfied	–
					Surveys				
					Focus groups				
					Document review				
					HIV testing data				

Vancouver STOP Project (26, 46)	Cross-sectional	Dental clinic	Not reported	22	Survey	–	Acceptability was high among clients tested	–	–

Vancouver STOP Project (26, 46)	Cohort	AIDS service organization, bathhouses, pride parade, parks, single-occupancy hotel rooms	Not reported	Not reported	Not reported	–	–	–	–

Veillette-Bourbeau (45)	Grounded theory	Community health center	MSM	10	Interviews	–	–	–	–
					Observation				
					Document review				

Wertheimer (43)	Participatory action research design	Sexual health clinics, community centers	Women	90	Interviews	–	–	–	–
					Surveys				

#### Acceptability

HIV POCT participant acceptability rates were measured in seven studies and ranged from 52 to 92%. Higher acceptability rates were reported among MSM (19). Participants also reported higher acceptability due to the availability of rapid HIV POCT results lessening wait times (20–23).

#### Feasibility

Three studies measured feasibility and determined that HIV POCT was feasible in hospitals (24), sexual health and HIV clinics (22), and outreach settings (25).

#### Linkages to Care

Linkages to care rates were 89% in one study (26) and 100% in two studies (19, 27). A third study demonstrated that peer HIV POCT helped relink 324 previously diagnosed individuals to care (28). While linkage to care rates are high, they reflect linkages to confirmatory HIV testing only, which is just one small step in the HIV care cascade; more information regarding linkage to counseling and retention to care is needed.

#### Loss to Follow-Up

Losses to follow-up were generally very low ranging from no loss (29) to a loss of 1.1% (30) and a loss of 3% (31).

#### Preferences

When compared to standard testing, participant preferences for HIV POCT ranged from 81.1 to 97%. A multiplex strategy in which individuals were tested for HIV, HCV, and other STIs was preferred by 97% (*n* = 109) of those enrolled in the study (24). Preferences for HIV POCT were reported by study participants in multiple settings including sexual health clinics (32, 33), primary care clinics (34), hospitals (24), community health centers (29), detention centers (35), and community-based organizations (31). For commercial sex workers in British Columbia, HIV POCT was preferred due to its flexibility and less invasive procedures (36).

#### Reach (To Those Who Have Never Tested)

Four programs were successful in reaching those who have never been tested. Forty-two percent of participants tested in two provincial correctional facilities in Ontario (20) and 61% of women tested in a correctional facility in Montréal, Québec were never-testers (35). Twelve and a half percent of commercial sex workers were also reached for the first time by trained outreach staff (36). Finally, 12.5% of participants reached in a sexual health clinic in Québec city had not previously been tested for HIV (22).

#### Reach (To Those Who Are Previously Tested)

In five studies (24, 27, 34, 37, 38), there were a large percentage of individuals who had previously been tested for HIV ranging from 50 to 96% of the total sample.

#### Returned Results (Confirmatory Testing)

Two studies measured rates of returned results. In one study, 100% of participants in a correctional facility in Montréal received test results (35). In the other study, 98% of testers at a sexual health clinic in Toronto received test results, of which 22 (1.5%) were reactive. Four of the 22 individuals who were tested with a rapid HIV test did not receive their results from confirmatory testing (37). Previous studies show that a high percentage of people with reactive (70–100%) results seek confirmatory testing (39).

#### Satisfaction

Satisfaction with HIV POCT was high among program participants with satisfaction levels between 96 and 100%. HIV POCT was reported to be less invasive, less stressful, and less painful than traditional models of HIV testing leading to increased satisfaction (22, 36, 40).

#### Sensitivity, Specificity, and Predictive Value of HIV POCT

Two studies (19, 41) compared the sensitivity of the HIV POCT to conventional HIV testing. In both studies, the sensitivity value of the HIV POCT was 100%.

Three studies (19, 31, 41) compared the specificity of the HIV POCT to conventional HIV testing. The specificity of the HIV POCT ranged from 99.8 to 99.9%.

Three studies (19, 31, 41) compared the positive predictive and negative predictive values of the HIV POCT with the predictive values of the standard serological test. The positive predictive value ranged from 66.7 to 96%, and the negative predictive value was 100% in all three studies.

## Discussion

Our scoping review investigated the utilization of HIV POCT in Canada. Our scoping review findings found evidence that HIV POCT has been implemented in five provinces and in a number of settings including community health centers and sexual health clinics, hospitals, primary care clinics, community organizations, correctional facilities, and outreach settings such as parks and gay pride parades. Moreover, HIV POCT programs have targeted the following populations: indigenous peoples, incarcerated individuals, LGBTQ individuals, MSM, people who use injection drugs, and pregnant women. The evidence in this review suggests that HIV POCT has broadened access to testing services for both those who have never tested and for return testers across much of Canada.

Overall, our scoping review found very high acceptance and satisfaction rates with HIV POCT programs in Canada. A large majority surveyed in these studies reported a preference for HIV POCT compared to conventional standard testing. Reasons commonly expressed to support these findings are that HIV POCT is more flexible, less invasive, and less stressful (due to a shortened wait period) than conventional testing. Moreover, losses to follow-up rates were generally very low for the HIV POCT programs identified in this review, while linkages to care rates were nearly perfect. In keeping with results from two other systematic reviews investigating the use and implementation of rapid HIV testing in North America (39) and among youth (42), the evidence in this review suggests that HIV POCT in Canada is feasible, preferred, and accepted by diverse populations.

The literature in this scoping review raises two important knowledge and policy gaps that should be addressed. First, HIV POCT services are not universally accessible across Canada (43, 44). In fact, there is little to no availability in the Northern Territories (Yukon, Northwest Territories, and Nunavut) and no availability in any of the four Atlantic Provinces. Second, HIV POCT is also unavailable in many rural and remote communities across Canada, some of which are First Nations, Inuit, and Métis communities. Further research and POCT services are required in these communities to understand how best to scale up HIV POCT in contexts that currently have very limited or no access to testing.

Despite these knowledge gaps, the evidence in this scoping review provides a number of actions to consider when implementing an HIV POCT program. First, program organizers must find qualified health-care professionals to offer HIV POCT (45), consider how to address confidentiality concerns, informed consent, and pretest counseling procedures (31, 33, 46), as well as ensure that confirmatory lab services are available and able to process additional POCT test kits (41). Program administrators also need to foster trusting relationships between participants and health-care providers (47) while providing multilingual programs and services that aim to enhance cultural safety (26, 36). Table 6 presents a summary of the implications for practice.

**Table 6:** **Implications for practice**.

1	HIV point-of-care testing (POCT) is more flexible, less invasive, and less stressful (due to a shortened wait period) than conventional testing
2	Program organizers must find qualified health-care professionals to offer HIV POCT
3	Concerns of confidentiality must be addressed
4	Confirmatory lab services must be available and able to process additional POCT test kits
5	Program organizers must develop trust between participants and health-care providers while providing multilingual and culturally safe services

Moreover, the approval and implementation of HIV POCT programs in any country will have a substantial impact on screening and public health programs as it raises questions related to costs, equitable access to testing services, uptake, streamlined counseling services, and timely linkages to care. Public health providers thinking about implementing HIV POCT programs can learn from the experiences of others who have already implemented these programs.

There are two limitations of our search strategy and evidence base that must be noted. First, while the analysis includes both peer-reviewed and grey literature sources, we relied primarily on electronic sources rather than both electronic and print sources, which may contribute to publication bias. However, we did search the reference lists of all included articles as well as contact well-known researchers who work on POCT in Canada. Moreover, based on the low quality assessment of the articles in this review, the findings, while overwhelming supportive of HIV POCT, are from a low evidence base. This evidence base is low as most studies were observational in nature, thus the results should be interpreted with appropriate cautions. These findings do suggest, however, that HIV POCT is widely accepted by the Canadian population, including among key populations, and has high satisfaction rates. However, we did not compare the impact of HIV POCT among different population or geographies, and our findings must be interpreted with appropriate considerations. In addition, narrowing the scope of this review to Canadian literature may affect the generalizability of this review; however, it increases its practicality by controlling for variations in national policies and regulations with respect to country-level HIV POCT. The practical and technical challenges identified at the local level in one nation may be beneficial to decision-makers in other places who are planning to scale up similar testing in other geographic locations or to prepare for implementation in other settings.

## Conclusion

In the process of this scoping review, we investigated 20 HIV POCT programs and 7 studies that elicited opinions including preferences for HIV POCT in Canadian settings. Our analysis focused on the utilization of HIV POCT including the feasibility, acceptability, satisfaction, preference for, and impacts of HIV POCT programs in Canada. Scoping reviews such as this one provide strong evidence of the benefits, reach, and acceptance of HIV POCT in Canadian provinces. They also identify important considerations for researchers, service providers, and policy makers when implementing new programs. The findings of this review will be useful to decision-makers both in Canada and globally as it distinguishes various barriers and enablers of successful implementation. These findings will help service providers anticipate potential challenges and maximize the benefits of HIV POCT in any setting.

## Author Contributions

A Minichiello, M Swab, MC, ZM, JG, A Maybank, AH, M Schwandt, SG, OH, and SA were involved in all stages of the project including design, screening, review, and analysis. M Swab designed the search strategy and coordinated the initial screening of all the references. SA, A Minichiello, M Swab, MC, A Maybank, and AH conducted screening, and data extraction. All authors contributed to and have approved the final manuscript. OH reviewed and provided comments for manuscript editing.

## Conflict of Interest Statement

The authors declare that the research was conducted in the absence of any commercial or financial relationships that could be construed as a potential conflict of interest.
